# Employment of Gun-Cotton in Cupping

**Published:** 1847-02

**Authors:** 


*Employment of Gun-Cotton in Cupping..*—The Prov. Med. and Surg. Journal of Dec. 9th contains the following announcement:—"It may be useful to know the value of gun-cotton in exhausting the air from cupping glasses; having so employed it myself on several occasions, I can recommend it as possessing a decided superiority over spirit; besides, its lightness and portability is an advantage at times. A very small portion is placed within the glass, and before a piece of lighted paper can be well introduced, from its highly inflammable nature it becomes ignited, imparting to the surface enclosed merely an agreeable warmth."

